# An advanced reference genome of *Trifolium subterraneum* L. reveals genes related to agronomic performance

**DOI:** 10.1111/pbi.12697

**Published:** 2017-03-23

**Authors:** Parwinder Kaur, Philipp E. Bayer, Zbyněk Milec, Jan Vrána, Yuxuan Yuan, Rudi Appels, David Edwards, Jacqueline Batley, Phillip Nichols, William Erskine, Jaroslav Doležel

**Affiliations:** ^1^ Centre for Plant Genetics and Breeding and Institute of Agriculture The University of Western Australia Crawley WA Australia; ^2^ School of Plant Biology and Institute of Agriculture The University of Western Australia Crawley WA Australia; ^3^ Institute of Experimental Botany Centre of the Region Haná for Biotechnological and Agricultural Research Olomouc Czech Republic; ^4^ Murdoch University Murdoch WA Australia; ^5^ Department of Agriculture and Food Western Australia South Perth WA Australia

**Keywords:** forage legumes, advanced reference assembly, BioNano, transcriptome, gene expression, Legume comparative genomics

## Abstract

Subterranean clover is an important annual forage legume, whose diploidy and inbreeding nature make it an ideal model for genomic analysis in *Trifolium*. We reported a draft genome assembly of the subterranean clover TSUd_r1.1. Here we evaluate genome mapping on nanochannel arrays and generation of a transcriptome atlas across tissues to advance the assembly and gene annotation. Using a BioNano‐based assembly spanning 512 Mb (93% genome coverage), we validated the draft assembly, anchored unplaced contigs and resolved misassemblies. Multiple contigs (264) from the draft assembly coalesced into 97 super‐scaffolds (43% of genome). Sequences longer than >1 Mb increased from 40 to 189 Mb giving 1.4‐fold increase in N50 with total genome in pseudomolecules improved from 73 to 80%. The advanced assembly was re‐annotated using transcriptome atlas data to contain 31 272 protein‐coding genes capturing >96% of the gene content. Functional characterization and GO enrichment confirmed gene expression for response to water deprivation, flavonoid biosynthesis and embryo development ending in seed dormancy, reflecting adaptation to the harsh Mediterranean environment. Comparative analyses across Papilionoideae identified 24 893 *Trifolium‐*specific and 6325 subterranean‐clover‐specific genes that could be mined further for traits such as geocarpy and grazing tolerance. Eight key traits, including persistence, improved livestock health by isoflavonoid production in addition to important agro‐morphological traits, were fine‐mapped on the high‐density SNP linkage map anchored to the assembly. This new genomic information is crucial to identify loci governing traits allowing marker‐assisted breeding, comparative mapping and identification of tissue‐specific gene promoters for biotechnological improvement of forage legumes.

## Introduction

Forage legumes are highly valued feed for extensive livestock production. There is an increasing interest worldwide in using annual forage legumes as cover crops to supply soil nitrogen (Sulas, 2005; Piano *et al*., 2010). Symbiotic nitrogen fixation in legumes leads to high protein fodder content and rejuvenated soils for a sustainable feed system. The clovers in particular are among the most effective to break the ‘infernal circle of the fallow’ a technique known to the Germans as ‘Besömmerung’ (Blanning, 2008). Subterranean clover (*Trifolium subterraneum* L.) makes the greatest contribution to livestock feed production and soil improvement in terms of total worldwide usage among annual clovers (McGuire, 1985), particularly in Australia, where it is sown over 29 mill. ha. The self‐reseeding ability and grazing tolerance of subterranean clover, even under suboptimal and variable environmental conditions (Nichols *et al*., 2013), contribute to its widespread distribution.

Subterranean clover is a diploid (2n = 2x = 16), predominantly inbreeding, annual species with a relatively small genome size of 540 Mbp (1C = 0.55 pg DNA; Vižintin *et al*., 2006) that can be readily hybridized, and exhibits wide diversity for both qualitative and quantitative agronomic and morphological characters (Ghamkhar *et al*., 2011). Within the genus *Trifolium*, it is established as a reference species for genetic and genomic studies. We reported a *de novo* draft genome assembly of the subterranean clover TSUd_r1.1, generated using a combination of long‐ and short‐read sequencing platforms (Figure S1) (Hirakawa *et al*., 2016). Genetic and genomic analyses of the internationally commercially important perennial legumes white clover and red clover are difficult, as they are outcrossing and have self‐incompatible fertilization, with white clover also being an allotetraploid (2n = 4x = 32) (Abberton and Marshall, 2005; Ghamkhar *et al*., 2011). As molecular markers developed for white and red clovers were readily transferable to subterranean clover (Ghamkhar *et al*., 2011), it is likely that subterranean clover QTLs and genes are applicable to these other species. Close synteny with the model legume, *Medicago truncatula*, also provides opportunities for genomic comparisons and the identification of candidate genes.

A variety of approaches are required for the *de novo* assemblies to improve draft genomes, and include a method developed by Dovetail Genomics for genome scaffolding using long‐range genomic information obtained by Chicago method (Putnam *et al*., 2016). In this study, we evaluated genome mapping on nanochannel arrays employing BioNano Irys^®^ system (www.bionanogenomics.com) to validate the initial draft assembly, anchor additional unplaced contigs and to resolve misassemblies, with the objective of improving the overall assembly coverage. BioNano genome mapping is a technique of optical mapping in which specific sequence motifs in single DNA molecules are fluorescently labelled. The labelled DNA molecules are loaded onto the IrysChip where they are electrophoretically linearized in thousands of silicon channels. Fluorescence imaging allows the construction of maps of the physical distances between occurrences of the sequence motifs (Lam *et al*., 2012). The aim was to advance the pseudomolecule assembly of the eight chromosomes of subterranean clover, based on the direct visualizations of sequence motifs on long single DNA molecules. Gene annotations for the *de novo* draft genome assembly of the subterranean clover TSUd_r1.1 were conducted using *in silico* automated Augustus and Maker pipelines only. This was compared with direct transcriptome analysis using whole genome RNA sequencing technology across five different tissues of subterranean clover to generate a valuable resource for the identification and characterization of genes and pathways underlying plant growth and development. This also provided a basis to investigate specific processes, biological functions and gene interactions for key agronomic traits.

## Results and discussion

### The advanced assembly

**Figure 1:**
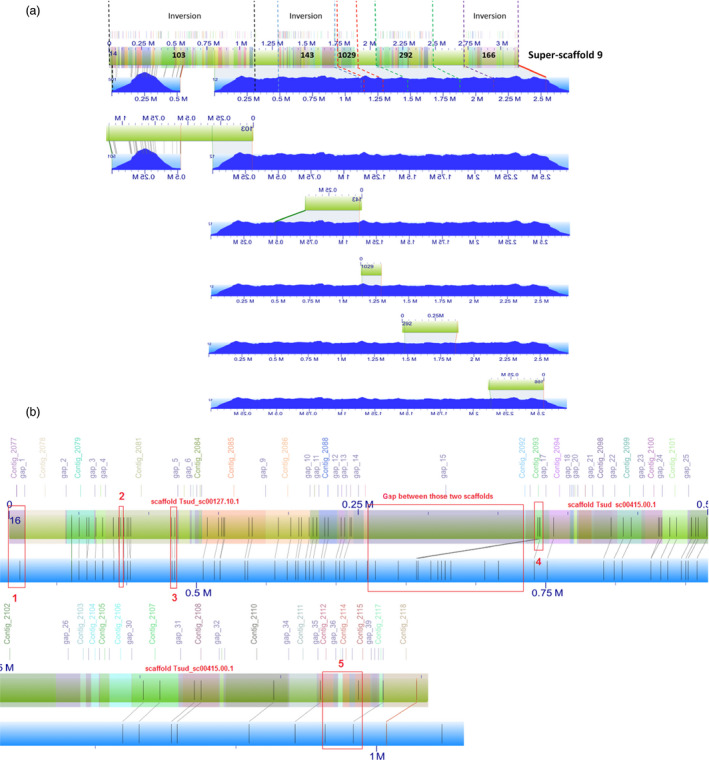
(a) *In silico* map of super‐scaffold 9 aligned to the BioNano optical maps (401× coverage). XMAP alignments for *in silico* map of sequence scaffolds 103, 143, 1029, 292 and 166 are shown. Consensus genome maps (blue with molecule map coverage shown in dark blue) align to the *in silico* maps of scaffolds (green with contigs overlaid as translucent coloured squares). An illustration of using BioNano optical maps to assist contig placement, scaffolding and inversion correction. Sequence scaffolds 103, 143, 1029, 292 and 166 were placed within super‐scaffold 9 by the optical maps and among these scaffolds; scaffolds 103, 143 and 166 were reversed in the super‐scaffold. (b) Illustration of misassemblies in the genome examined using BioNano optical maps. The super‐scaffold (colourful bar) contains scaffold Tsud_sc00127.10.1, scaffold Tsud_sc00415.00.1 and a gap between them. Compared with the optical map (blue bar), there are more *Bsp* QI restriction cut sites (displayed as straight lines) in the super‐scaffold at positions 2; there are some *Bsp* QI restriction cut sites missing at positions 1. At positions 3, 4 and 5, the super‐scaffold does not consist of BioNano consensus map. All those discordances can be examined in detail in the corresponding contigs or gaps (indicated above the super‐scaffold).

The first draft genome assembly of the Australian subterranean clover variety, *cv*. Daliak, covers 85.4% of the estimated genome in eight pseudomolecules of 401.1 Mb length and was constructed using a linkage map consisting of 35 341 SNPs (Hirakawa *et al*., 2016). In this study, we evaluated genome mapping *via* scaffolding using *de novo* physical maps generated using the BioNano Genomics (BNG) Irys platform to improve the genome assembly. A total of 221.7 Gb (401× genome coverage) of filtered data (molecules >150 kb) was generated on the Irys instrument. After filtering out low‐quality single molecules, a total of 188.5 Gb (341× genome coverage) of data was included in the final BioNano‐based *de novo* physical map assembly. This physical map assembly consisted of 309 075 individual molecules and 468 consensus maps that spanned 512 Mb (93% genome coverage) with N50 of 1.4 Mb. Multiple contigs (264) from the first draft assembly coalesced into 97 super‐scaffolds (containing 43% of the total genome captured) (Figure 1). In the advanced assembly (Tsub_Refv2.0), the size of sequences longer than >1 Mb increased dramatically from 40 to 189 Mb. This resulted in a 1.4‐fold increase in the N50 with the total percentage of genome captured in pseudomolecules improving from 73 to 80% with a substantial reduction of sequence gaps (Table 1).

**Table 1:** BioNano genome mapping statistics (*cv*. Daliak)

N Genome Maps	468
Total Genome Map Len (Mb)	512.439
Avg. Genome Map Len (Mb)	1.095
Genome Map N50 (Mb)	1.408
Molecule Stats	
Contig Coverage (x)	367.81
Molecules Aligned to the first draft genome assembly TSUd_r1.1	
N mol align	30 9075
Mol fraction align	0.37
Tot align len (Mb)	59930.8
Avg align len (kb)	193.9

In this study, the BNG Irys platform provided affordable, high‐throughput physical maps of improved contiguity to validate the draft assembly (Hirakawa *et al*., 2016) generated across a combination of long‐read and short‐read platforms and extended scaffolds. In contrast to alternative, sequencing‐based approaches (e.g. Chicago method of Dovetail Genomics, GemCode Technology of 10× Genomics, or Hi‐C), it enables a highly accurate sizing of gaps in sequence assemblies and provides a real picture of genomic regions intractable to current sequencing technologies, such as long arrays of tandem repeats (Staňková *et al*., 2016). Thus, combinations of platforms are recommended and no one platform is a perfect technology to use in answering every research question (Chaney *et al*., 2016).

### The transcriptome ATLAS and gene features

The identification and annotation of expressed genes within the *T. subterraneum* genome assembly used high‐throughput whole genome RNA sequencing analyses to predict a total of 32 333 transcripts for 31 272 protein‐coding genes, with evidence for their expression across five different tissue types. The process‐involved annotations that combined evidence from transcriptome alignments obtained from protein homology and *in silico* gene prediction derived from different tissue types (roots, stem and peduncles, leaf and petioles, flowers and developing seeds) of *cv*. Daliak (Table S1). Phytozome and TrEMBL were the most informative databases for assigning functional annotations to subterranean clover proteins, with 29 157 (90.2%) and 29 278 (89.9%) proteins annotated, respectively (Table S2). In the draft TSUd_r1.1, the presence of 42 706 genes was predicted from *in silico* evidence using homology studies, domain searches and *ab initio* gene predictions. The reduction in genes annotated in the present study was achieved because the latter computational methods are unable to provide definitive evidence about which genes are actually expressed (Guigó *et al*., 2000; Wheelan and Boguski, 1998).

**Figure 2:**
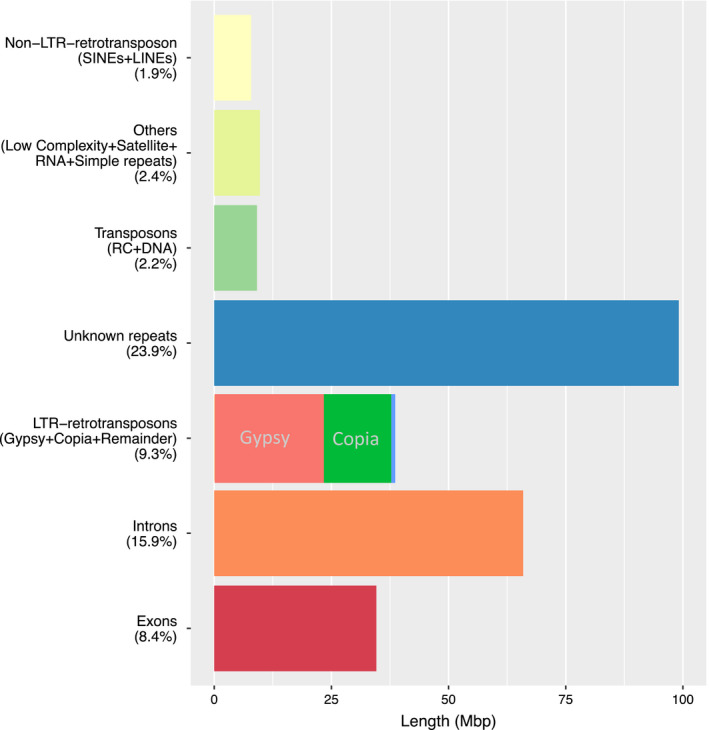
Functional composition of the assembled reference sequence *Trifolium subterraneum L*. genome.

Repetitive element analysis predicted that 64% of the genome comprises repeat sequences, of which 15.9% and 8.4% are within introns and exons, respectively. About a quarter (23.9%) of repetitive elements were classified in the unknown zone. Like most eukaryotes, transposable elements (TEs) were most commonly long terminal repeats (LTR) retrotransposons (9.3%), with *Gypsy*‐like elements as the most frequently classified retrotransposons. Other TEs such as DNA, rolling circle (RC) and non‐LTR long retrotransposons such as long interspersed nuclear elements (LINEs) and short interspersed nuclear elements (SINEs) comprised a relatively small proportion (2.2% and 1.9%, respectively) (Figure 2; Table S3). Non‐coding RNA was estimated to comprise 0.12% of the genome, the majority being ribosomal RNA (0.02%) and transfer RNA (0.01%), with predicted snRNA and miRNA representing 0.01% and 0.07%, respectively (Table S4).

To test the quality of the advanced genome assembly and re‐annotation, we conducted a CEGMA analysis (Parra *et al*., 2007) to identify the presence of core eukaryotic genes. From the core set of 248 eukaryotic genes, there were 240 complete and 247 partial genes present in the advanced assembly, representing 96.8% and 99.6% [in comparison with the 95.6% and 98.0% for the draft assembly TSUd_r1.1] genes of the core set, respectively (Table S5).

### Functional characterization and GO enrichment analyses of the advanced genome

The advanced assembly was functionally characterized by assigning gene ontology (GO) terms to proteins by manually transferring the GO terms for Swiss‐Prot IDs using the UniProt‐GOA database (Huntley *et al*., 2015). All these protein‐coding genes/transcripts were grouped into the three main GO categories: biological processes, molecular function and cellular components. A total of 21 210 (65.6%) of the subterranean clover protein‐coding genes/transcripts were assigned GO terms (Table S1). Of the 131 324 GO terms identified, 5,648 appear only once. There are 1013 GO terms appearing between 50 and 200 times with a total sum of 35 383 (Figure S2).

Among the 5648 GO terms appearing once, the top five most highly represented groups in the biological processes category were genes/transcripts associated with signal transduction, response to abscisic acid, cellular response to DNA damage stimulus, methylation and response to water deprivation (Table S6, for enriched cellular components and molecular functions see Tables S7 and S8, respectively). More detailed classification of the biological process GO category also showed enrichment in comparison with whole UniProt for response to cold and oxidative stress, flower development, flavonoid biosynthesis and embryo development ending in seed dormancy as highly represented groups. Overall, this functional characterization and GO enrichment analysis confirmed gene expression for response to water deprivation, cold and oxidative stress, flavonoid biosynthetic process and embryo development ending in seed dormancy reflecting the adaptation of subterranean clover to the harsh Mediterranean environment.

### Global gene expression trends

**Figure 3:**
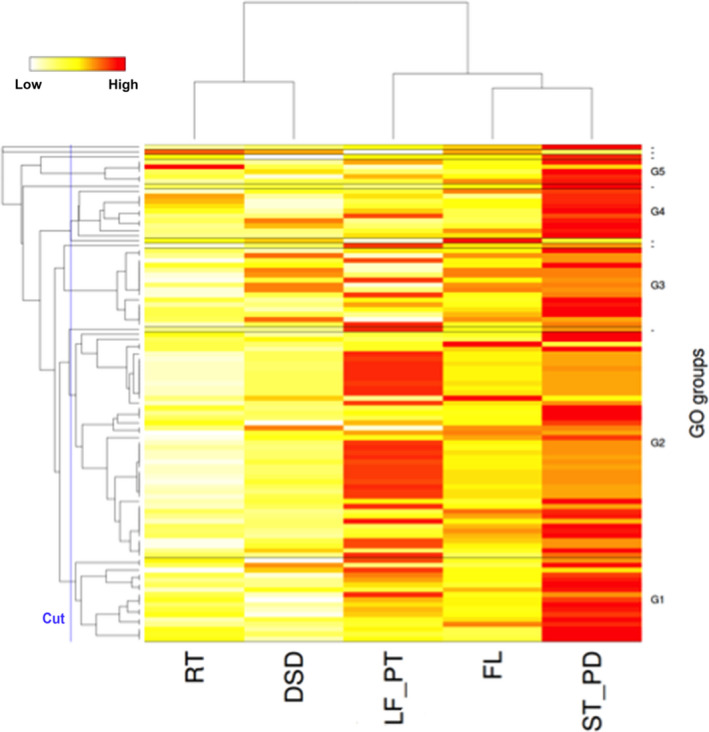
Heat map showing hierarchical clustering of tissues based on global gene expression and GO terms. Clustering was based on 100 genes with highest sum of all TPM (transcripts per million) of the five tissue types. The dendrogram of the selected genes [vertical axis] is visualized along with the expression patterns that are used to cluster the five tissue samples, i.e. root (RT), developing seed (DSD), leaf and petioles (LF_PT), stem and peduncles (ST_PD) and flower (FL) [horizontal axis]. There are five GO‐based clusters named G1…G5 that contain more than one gene. The GO clusters are separated by a horizontal bar in the heat map. Genes without annotations are omitted from the heat map. Highly expressed genes are shown with red colour and lower expressed genes with white or yellow colour, respectively.

To investigate the representation of genes among the five tissue types, hierarchical clustering of tissues, based on global gene expression and GO terms, was performed on 100 genes with the highest sum of all transcripts per million (TPM). The biological identity of the tissues was clearly reflected in the analysis by hierarchical clustering of the RNA‐Seq‐based transcriptome (Figure 3; Table S9). Predicted genes showed substantial variation in their expression over tissues, reflecting tissue‐specific biological activities. For instance, in leaf and petiole tissue GO‐based clusters G1 and G2 were enriched and genes‐encoding proteins involved in photosynthesis, binding and chloroplast thylakoid membranes were over‐represented consistent with the specialized biological function of these tissues. Likewise, the root transcriptome profile showed enriched expression for stress and defence responses with genes encoding for cysteine‐type endopeptidase inhibitor activity. However, the G3 cluster indicates commonalities in the transcriptome of all above ground vegetative tissue types and showed enrichments for translational and structural ribosomal pathways.

For tissues with predominance of a specific biological activity, a large proportion of reads may have represented abundantly expressed genes and, therefore, deeper sequencing may be needed to detect genes with relatively low expression levels. However, the high correlation between biological replicates and the clustering of tissues based on their biology indicates that the sampling depth in this study is sufficient to draw inferences about the transcriptome.

### Dynamic spatial gene expression analysis

**Figure 4:**
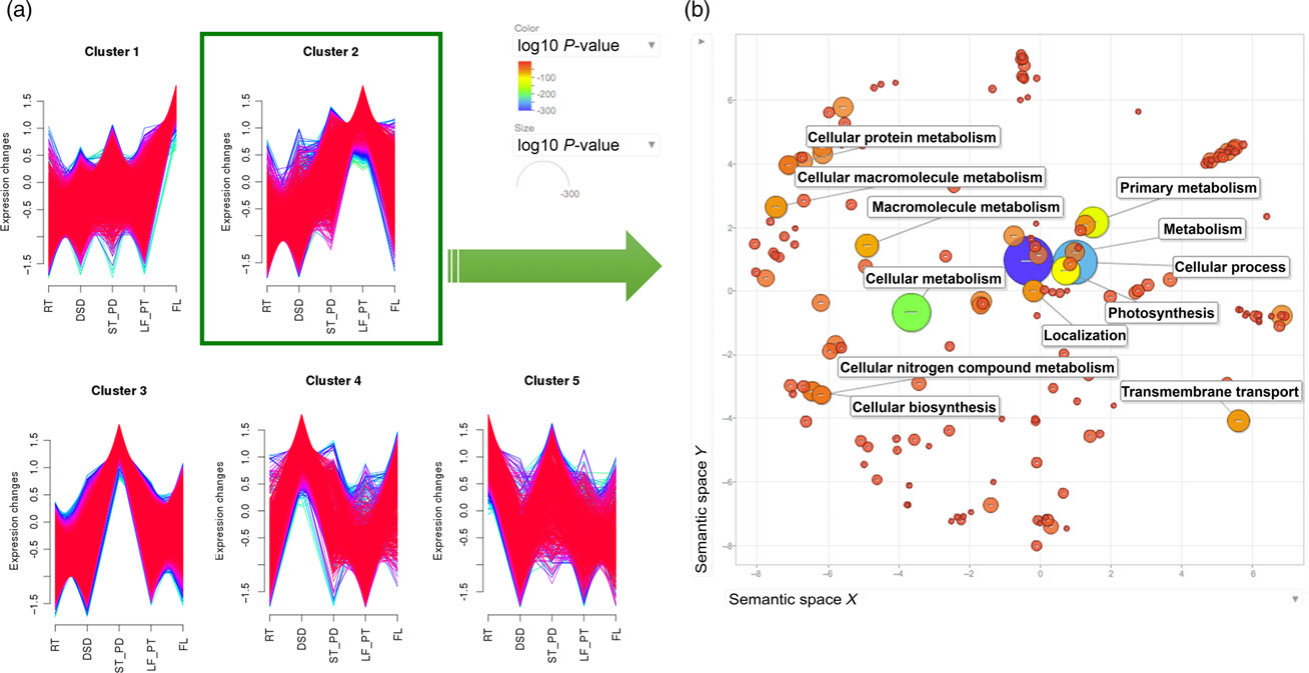
Dynamic tissue‐specific gene co‐expression clusters. (a) Five clusters were generated using Fuzzy C‐means soft clustering algorithm implemented in Mfuzz. Data points on the X‐axis represent root (RT), developing seed (DSD), stem and peduncles (ST_PD), leaf and petioles (LF_PT), flowers (FL) tissues, respectively. The Y‐axis represents gene expression values, where gene expression values were standardized to have zero mean and one standard deviation. (b) The scatterplot generated using REVIGO web browser shows the cluster representatives (i.e. terms remaining after the redundancy reduction) for leaf and petiole tissue‐enriched Cluster 2 biological processes in a two‐dimensional space derived by applying multidimensional scaling to a matrix of the GO terms’ semantic similarities. Bubble colour indicates the *P*‐value (legend in upper left‐hand corner); size indicates the frequency of the GO term in the wholeUniProt GOA database (bubbles of more general terms are larger).

The tissues sampled in the RNA library preparations allow the determination of dynamic spatial gene expression and characterization. A comparative gene expression analysis between five tissue types (Table S1), using the advanced genome assembly (Tsub_Refv2.0) as the reference, revealed tissue‐specific functional diversification of paralogous genes. Specific gene expression in each of the five different tissues was indicated by the comparison of global gene expression across tissues. To explore gene expression by tissue‐type further, differentially expressed genes were identified in all pairwise tissue‐type comparisons. These differentially expressed genes were then used to identify clusters of co‐expressed genes, which represent spatially enriched expression patterns. Such co‐expression clusters were identified by the Mfuzz package (Futschik and Carlisle, 2005) using the soft clustering approach with fuzzy c‐means algorithm. This analysis generated five clusters, which revealed dynamic gene expression patterns across the five tissue types (Figure 4a; Table S10).

Cluster 1 included genes with the highest expression in floral tissue (Figure 4b). Among genes in this cluster several are of interest. For example, this cluster includes many genes defining floral organ identity such as floral homeotic protein AGAMOUS, APETALA 2, PMADS 2 and DEFICIENS. In addition, Cluster 1 also included chromatin structure‐remodelling complex protein SYD, MADS‐box protein CMB1 and many chloroplast and carotenoid genes. Leaf‐enriched Cluster 2 included transcripts‐encoding enzymes involved in photosynthesis, Rubisco and oxygenase activity in addition to many chloroplast and plastid‐regulating genes. Cluster 3 contained genes with high expression in stem and peduncle tissue, and included genes/transcripts‐encoding enzymes involved in nutrient transport, structural growth with a high activity of various receptor kinases, expansins and transferases. Developing seed‐enriched Cluster 4 showed genes genes/transcripts involved with embryogenesis and growth. Cluster 5 enriched with root tissue gene expression profiles included different nodulins, binding proteins, MYB‐transcription factors and both stress‐ and defence‐related genes.

Genes within different clusters are potential sources of tissue‐specific promoters. For example, to improve forage nutritional quality by isoflavonoid biosynthesis or digestibility through processes such as cell wall loosening and lignin biosynthesis, promoters specific to leaf tissue are required. Options for transformation have recently broadened to include genome‐editing tools such as CRISPR‐cas9 (Rani *et al*., 2016).

### Functional classification of the tissue‐enriched gene expression clusters

Genes representing tissue‐enriched clusters were subjected to GO enrichment analysis to further characterize and identify over‐represented functional groups in subterranean clover using REVIGO web server (Supek *et al*., 2011) to graphically represent the results. The scatterplot depicting leaf‐enriched cluster (Cluster 2) showed enrichment for genes associated with photosynthesis, transport and response to stimulus (Figure 4b; Table S11). The flower‐enriched cluster (1) showed a concentration of genes associated with the processes of nitrogen compound cellular biosynthesis, multicellular organismal organization and development (Table S12). The stem‐ and peduncle‐enriched cluster (3) displayed a high representation of genes associated with response to stimulus, stress, oxidation‐reduction process, transport and signal transduction. The root‐enriched cluster (5) had a high representation of genes associated with response to stress, stimulus, nutrient levels, transport and nitrate metabolism. Clearly, the GO enrichment analyses of tissue‐enriched clusters showed over‐representation of predicted classes of genes in different tissue types and thereby validated our tissue‐enriched gene co‐expression clusters. Additionally, putative genes‐encoding enzymes in subterranean clover were assigned to various pathways in the KEGG database using BLASTKOALA (Kanehisa *et al*., 2016). This analysis distributed 4,094 genes‐encoding enzymes (12.7%) into 133 different KEGG pathways (Figure S3; Table S13).

### Papilionoideae whole genome phylogenetic analyses

**Figure 5:**
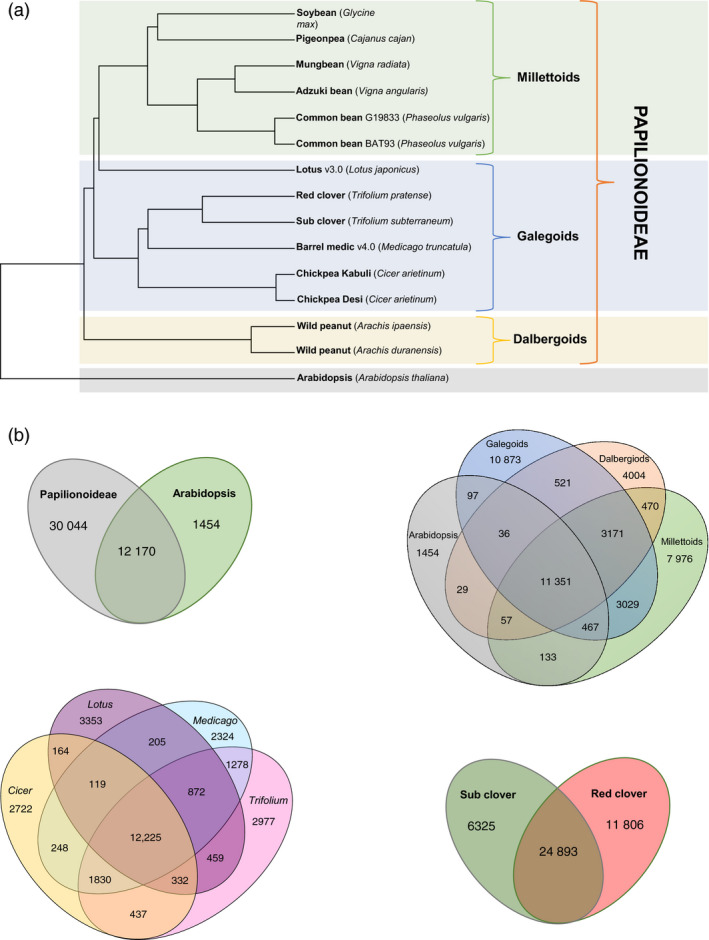
(a) Phylogenetic tree using the published predicted protein sequences for 13 legume genomes including the newly annotated *Trifolium subterraneum* L. and *Arabidopsis thaliana*. Mash v1.1 (preprint: http://biorxiv.org/content/early/2016/04/19/029827) was used to calculate a distance matrix and pairwise mutation distance estimation using the published whole genome sequences for all the legumes except the transcriptome assembly for *Cajanus cajan* as a whole genome assembly was not available. The phylogeny was constructed using UPGMA as implemented in the R‐package ‘phangorn’ v2.0.3. (b) Shared and unique gene clusters in A) Papilionoideae species and *A. thaliana*; B) Millettioid, Galegoid and Dalbergioid clade or and *A. thaliana*; C) Lotus, Trifoliums, Medicago and Cicers; D) Shared and unique genes in sub‐clover and red clover.

Comparative analyses across the Papilionoideae using the published whole genome sequences for 13 legumes and the Tsub_Refv2.0 (Figure 5a and b; Table S14) revealed an overlap of 12 170 orthologous gene clusters between Papilionoideae and *Arabidopsis thaliana* as the outgroup species. Within the Papilionoideae, the number of orthologous gene clusters specific to galegoid (*Lotus japonicus, Trifolium pratense, T. subterraneum, M. truncatula* and *Cicer arietinum*), millettioid (*Glycine max, Cajanus cajan, Vigna radiata, V. angularis* and *Phaseolus vulgaris*) and dalbergoid (*Arachis ipaensis* and *A. duranensis*) was 10 873, 7976 and 4004, respectively. There were 3171 orthologous gene clusters in common among galegoid, millettioid and dalbergoid species.

**Figure 6:**
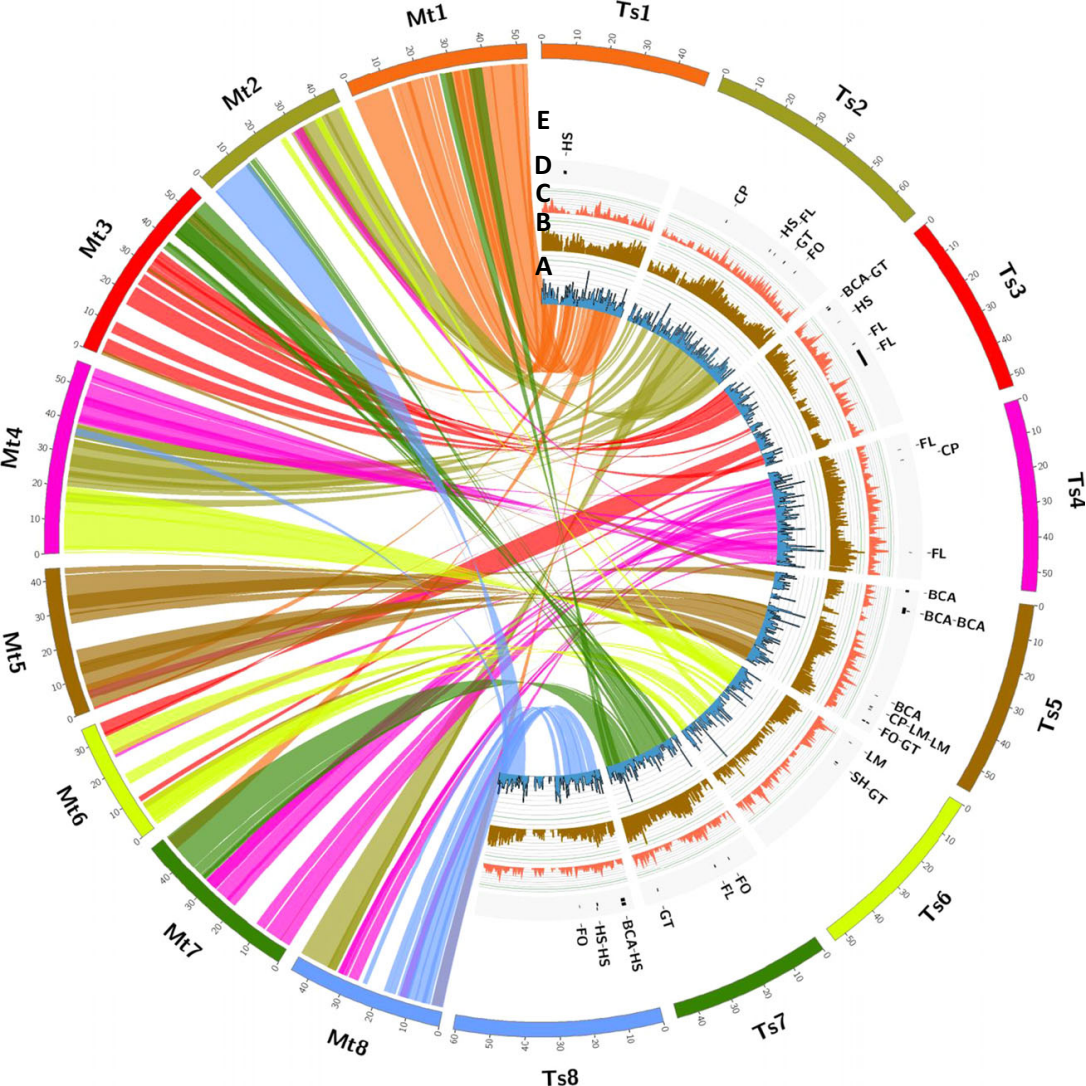
Graphical view of the genome structure of *Trifolium subterraneum L*. A) Syntenic relationship between *Medicago truncatula* (left) and *T. subterraneum* (right) with synteny links and synteny density histograms; B) *T. subterraneum* gene densities C) *T. subterraneum* SNP densities; D) *T. subterraneum* QTLs mapped for important traits using the high‐density SNP linkage map E) Names for the QTLs mapped for important traits using the high‐density SNP linkage map [FL Flowering time, LM Leaf marks, CP Calyx pigmentation, SH Stem Hairiness, FO Formononetin, GT Genistein, BCA Biochanin A and HS Hardseededness].

To find *Trifolium*‐specific genes among the galegoids, we identified 18 131 genes representing 24.3% of the total 74 556 genes using BLAST searches (Table S15). This proportion is higher than reported in chickpea (10%; Garg *et al*., 2011; Jain *et al*., 2013) Arabidopsis (4.9%; Lin *et al*., 2010) and rice (17.4%; Campbell *et al*., 2007). *Trifolium*‐specific gene clusters are a source of unique genes controlling important traits as drought tolerance, and disease resistance. Within the genus *Trifolium,* similar BLAST searches identified 6,325 (19.6%) and 11 806 (28.0%) genes as subterranean‐clover‐specific and red‐clover‐specific genes, respectively (Tables S16 and S17). These candidate subterranean‐clover‐specific genes could be mined further for such key traits as geocarpy and other factors related to the grazing tolerance of subterranean clover. Examining orthologous gene clusters provides an important foundation for comparative biology and functional inference in subterranean clover, because genes with simple orthologous relationships often have conserved functions, whereas genes duplicated more recently relative to speciation often underlie functional diversification. Comparative genomic analysis of the advanced subterranean assembly with the model legume, *Medicago truncatula*, showed close synteny and extensive collinearity of large sequence blocks (Figure 6). This provides opportunities for genomic comparisons and translation to identify candidate genes for traits of interest in the two species.

### Marker‐trait association studies

Phenotypic information for eight important traits described by Ghamkhar *et al*. (2011) (flowering time, hardseededness, leaf marks, calyx tube pigmentation, stem hairiness and the isoflavonoids, formononetin, genistein, and biochanin A) was associated with specific regions in the revised high‐density SNP linkage map (Tables S18 and S19) previously described by Hirakawa *et al*., 2016;. Significant associations were then mapped onto the advanced assembly (Tsub_Refv2.0) to identify possible candidate genes (Figure 6; Table 2; Table 3). The effective anchoring of sequence scaffolds onto the high‐density SNP linkage map in a high‐quality chromosome‐level genome assembly was a major factor in the identification of loci governing key traits. For example, QTLs for leaf marks (LM), calyx pigmentation (CP) and the isoflavonoids [formononetin (FO), genistein (GT) and biochanin A (BCA)] were found to map adjacently to a region on Chr 5. The linkage between these traits was demonstrated by Francis and Millington (1965), who used mutagenesis on *cv*. Geraldton, which has a high formononetin content, a prominent leaf mark and anthocyanin pigmentation of leaves, calyx tubes, stipules and stems to produce the low formononetin *cv*. Uniwager, which concomitantly lost its leaf mark and anthocyanin pigmentation. Co‐localization of the QTLs identified for the traits in the present study explains the observation of breeders that isoflavone content is linked to pigmentation traits (leaf and calyx) (Figure 6; Table 3) (Francis and Millington, 1965). This information illustrates links between the new assembly, the high‐density linkage map and key quantitative traits that can assist future marker‐assisted selection and comparative mapping with other species to improve forage legumes and increase livestock productivity.

**Table 2:** Assembly and predicted gene statistics for advanced Tsub_Refv2.0 assembly using RNAseq supported data in comparison to the first draft genome assembly TSUd_r1.1

	TSUd_r1.1 contigs	TSUd_r1.1_pseudomolecules	Tsub_Refv2.0 scaffolds	Tsub_Refv2.0_pseudomolecules
Number of Sequences	27 424	1702	27 257	1545
Maximum length (bases)	2 878 652	63 731 624	7 089 608	67 952 282
Minimum length (bases)	300	42 658 284	300	46 363 611
Average length (bases)	17 205	50 143 517	19 481	55 397 738
Total % of genome captured in pseudomolecules (bp)^a^	–	72.62	–	80.23
N50 length (bases)	287 605	–	410 493	–
Number sequences >100 Kbp	1 302	–	1 135	–
Size of sequences >100 Kbp	386 665 122	–	445 838 546	–
Number sequences >1 Mbp	31	–	86	–
Size of sequences >1 Mbp	40 489 272	–	189 281 499	–
Number of sequences contained in super–scaffolds	–	–	97^b^	–
Length of sequences contained in super–scaffolds	–	–	193 110 501	–
GC%	33.3	–	33.3	–
Number of predicted genes	42 706	–	32 333	–
Total length of predicted genes (bp)	47 965 017	–	34 758 167	–
Average length of predicted genes (bp)	1123	–	1075	–
Max length of predicted genes (bp)	15 417	–	15 309	–
Min length of predicted genes (bp)	150	–	201	–
N50 length of predicted genes (bp)	1548	–	1437	–

a Estimated genome size 552.4 Mb.

b Consists of 264 contigs from TSUd_r1.1.

**Table 3:** Major QTLs and candidate genes mapped for the important traits using the high‐density SNP linkage map anchoring to the advanced Tsub_Refv2.0 assembly

Trait Name	Trait ID	Chr	Left Marker	Right Marker	LOD	PVE (%)	Candidate genes
Flowering time	FL	3	Tsud_sc00634.00_113902	Tsud_sc01407.00_29664	36.36	61.94	87
		3	Tsud_sc00557.00_23548	Tsud_sc01682.00_2842	4.78	4.93	6
		2	Tsud_sc01286.00_17178	Tsud_sc01608.00_15349	3.25	3.14	5
		4	Tsud_sc00001.20_668469	Tsud_sc00001.20_681817	3.10	2.99	2
		4	Tsud_sc00277.00_152530	Tsud_sc00277.00_243760	2.76	2.43	8
		7	Tsud_sc00124.00_567396	Tsud_sc00124.00_311320	2.98	2.85	28
Leaf marks	LM	5	Tsud_sc01769.00_33298	Tsud_sc01806.00_29808	91.74	52.58	3
		5	Tsud_sc00041.00_165499	Tsud_sc00041.00_347862	25.67	8.90	16
		6	Tsud_sc00011.20_420838	Tsud_sc00011.20_536648	2.76	0.61	15
Calyx pigmentation	CP	5	Tsud_sc00695.00_95603	Tsud_sc00094.10_42823	111.79	96.88	13
		4	Tsud_sc00164.00_12669	Tsud_sc00164.00_104886	2.96	0.50	6
		2	Tsud_sc00213.10_37103	Tsud_sc00274.00_337396	2.70	0.48	3
Stem hairiness	SH	6	Tsud_sc00057.00_9596	Tsud_sc00057.00_401981	136.43	98.71	30
Formononetin	FO	5	Tsud_sc00724.00_157584	Tsud_sc00955.00_89974	17.25	25.53	30
		2	Tsud_sc00651.20_25763	Tsud_sc01310.00_44071	12.43	15.53	5
		7	Tsud_sc00459.00_51828	Tsud_sc00459.00_195702	7.42	9.03	13
		8	Tsud_sc00629.00_42778	Tsud_sc00629.00_69362	4.12	4.74	3
Genistein	GT	2	Tsud_sc01915.00_18283	Tsud_sc00906.00_202766	5.80	12.07	19
		3	Tsud_sc00026.00_688983	Tsud_sc00178.00_122069	5.57	11.50	34
		7	Tsud_sc00024.00_176871	Tsud_sc00024.00_366227	5.05	10.00	25
		6	Tsud_sc01326.00_38538	Tsud_sc01503.00_12748	3.12	6.17	0
		5	Tsud_sc00724.00_157584	Tsud_sc00955.00_89974	2.75	5.38	30
Biochanin_A	BCA	3	Tsud_sc00211.20_34823	Tsud_sc00026.00_63478	9.60	23.45	19
		8	Tsud_sc00654.20_18111	Tsud_sc01006.00_41002	4.53	10.40	30
		5	Tsud_sc00222.00_346664	Tsud_sc00242.00_222297	2.92	6.22	37
		5	Tsud_sc00285.00_86451	Tsud_sc00285.00_325955	2.76	5.84	10
		5	Tsud_sc02041.00_8358	Tsud_sc00674.20_21693	2.70	5.76	1
		5	Tsud_sc00073.20_492966	Tsud_sc00076.10_146128	2.64	5.60	87
Hardseededness	HS	1	Tsud_sc00068.20_1257208	Tsud_sc00855.00_29720	9.29	36.24	80
		3	Tsud_sc00009.30_108575	Tsud_sc00009.30_115638	2.76	6.62	2
		8	Tsud_sc00654.20_18111	Tsud_sc01006.00_41002	2.70	6.50	30
		8	Tsud_sc00309.00_532001	Tsud_sc00204.10_27738	2.58	6.46	10
		8	Tsud_sc00204.10_27738	Tsud_sc00431.00_91208	2.61	6.29	12
		2	Tsud_sc00682.00_120236	Tsud_sc01560.00_10224	2.55	6.16	6

## Conclusion

To improve the draft assembly of subterranean clover, the BNG Irys^®^ system provided affordable, high‐throughput physical maps of high contiguity to validate the draft and extend existing scaffolds. Effective anchoring of sequence scaffolds to genetic linkage groups, coupled with the use of the BioNano system, resulted in a high‐confidence chromosome‐level genome assembly. Tissue‐specific transcript profiling with RNA‐Seq technology delivered gene expression data of high value for gene annotation of the assembly (Tsub_Refv2.0) and transcriptional dynamics to understand tissue‐specific pathways. This new genomic information is the key to identifying loci governing traits that allow marker‐assisted breeding in subterranean clover for comparative mapping with other species and the identification of tissue‐specific gene promoters for biotechnological improvement of forage legumes.

## Experimental procedures

### Plant materials

Suspensions of intact cell nuclei were prepared according to Vrána *et al*. (2016). Briefly, mature dry seeds of subterranean clover (*Trifolium subterraneum* L.) *cv*. Daliak (approx. 20 g) were germinated on moistened paper towels in the dark at 25° ± 0.5 °C until roots achieved 2–3 cm in length. Roots were cut to 1 cm from the apex and transferred into 25 mL formaldehyde fixative (2% v/v) for 20 min at 5 °C, followed by three 5‐min washes in Tris buffer. Finally, the root tips (approx. 40) were cut, transferred into 1 mL IB buffer (Šimková *et al*., 2003) and homogenized using a mechanical homogenizer (blender) at 13 000 RPM for 18 s. The crude homogenate was filtered through 50‐μm (pore size) nylon mesh and stained with DAPI (2 μg/mL final concentration). A total of fifteen samples were prepared.

For transcriptome work, subterranean clover *cv*. Daliak plants were sown on 1 May 2015 in the field at Shenton Park, Western Australia (31^o^57′S, 115^o^50′E). Five different tissue types (roots, stem and peduncles, leaf and petioles, flowers and developing seeds) were harvested from a single Daliak plant on 24 September, when it was flowering and setting seeds. All samples were taken between 10.00 am and noon to eliminate any diurnal variations.

### Preparation of high molecular weight (HMW) DNA for BioNano mapping

Cell nuclei were purified by a FACSAria II SORP flow cytometer and sorter (BD Biosciences, San Jose, CA) equipped with UV laser for DAPI excitation. Populations of G_1_ nuclei were sorted in batches of 700 000 into 1.5‐mL polystyrene tubes containing 660 μL IB buffer. In total, four batches of nuclei were sorted and each batch was used for preparation of one 20‐μL agarose miniplug. DNA embedded in miniplugs was purified by proteinase K (Roche, Basel, Switzerland) treatment according to Šimková *et al*. (2003). The miniplugs were washed four times in wash buffer (10 mm Tris, 50 mm EDTA, pH 8.0) and five times in TE buffer (10 mm Tris, 1 mm EDTA, pH 8.0) and then melted for 5 min at 70 °C and solubilized with GELase (Epicentre, Madison, WI) for 45 min. A drop dialysis against TE buffer (Merck Millipore, Billerica, MA) was performed for 90 min to purify DNA from any residues and subsequently quantified using a Quant‐iT^TM^ PicoGreen^®^ dsDNA assay (Thermo Fisher Scientific, Waltham, MA).

### RNA extractions and library preparation

Total RNA from all the tissue samples (Figure S4) was extracted using the Spectrum™ Plant Total RNA Kit (Sigma‐Aldrich, USA) following the manufacturer's instructions. Aliquots of purified RNA were stored at −80 °C. The concentration of RNA was confirmed using Qubit fluorometer with Qubit RNA assay kit (Life Technologies, Australia). The integrity of total RNA was determined by electrophoretic separation on 1.2% (w/v) denaturing agarose gels. Sequencing libraries were constructed using 500 ng of total RNA with a TruSeq^®^ Stranded Total RNA Sample Prep Kits with Ribo‐Zero (Illumina, San‐Diego, USA) following the manufacturer's instructions. Library concentrations were measured using a Qubit fluorometer with Qubit dsDNA BR assay kit (Life Technologies, USA) and Agilent high‐sensitivity DNA chips (Agilent Technologies, USA). The amplified libraries were pooled in equimolar amounts, and quality was assessed with Agilent high‐sensitivity DNA chips (Agilent Technologies, USA). Paired‐end 100‐bp x 2 sequencing was performed with HiSeq2000 (Illumina, San‐Diego, USA).

### BioNano mapping

The genome sequence of subterranean clover (TSUd_r1.1) (Hirakawa *et al*., 2016) was analysed with Nickers software to assess the frequency of recognition sites for four different nicking enzymes (*Nt.BbvC1*,* Nt.BsPQ1*,* Nb.bsm1* and *Nb.BsrD1*). The optimal labelling frequency was calculated for endonuclease *Nt.BsPQ1* (7.6 sites/100 kb). DNA was then processed using NLRS protocol using the IrysPrep^®^ Reagent Kit (BioNano Genomics, San Diego, CA) following manufacturer's instructions. DNA was nicked using 8U of *Nt.BspQ1* (New England BioLabs, Beverly, MA) for two hours at 37 °C in NEBuffer 3. The nicked DNA was labelled with a fluorescent‐dUTP nucleotide analogue using Taq polymerase (New England BioLabs) for one hour at 72 °C. After labelling, the nicks were ligated with Taq ligase (New England BioLabs) in the presence of dNTPs for 30 min at 37 °C. The backbone of the labelled DNA was stained with IrysPrep^®^ DNA Stain (BioNano Genomics). The NLRS DNA concentration was measured again with the Quant‐iT^TM^ PicoGreen^®^ dsDNA assay.

Labelled and stained DNA was loaded on the Irys chip, and four consecutive runs were performed (each run consisting of 30 cycles). A total of 489.8 Gb data were generated, of which 221.7 Gb exceeded 150 kb, the threshold for map assembly. These filtered data (>150 kb), corresponding to 401× coverage of the subterranean clover genome, were compiled from 994 895 molecules with N50 of 215.4 kb. *De novo* assembly of the BioNano map was performed by a pairwise comparison of all single molecules and graph building (Cao *et al*., 2014). A *P*‐value threshold of 1e^−9^ was used during the pairwise assembly, 1e^−10^ for extension and refinement steps and 1e^−15^ for a final refinement.

### Stitch: super scaffolding and correcting potential misassemblies

The complete pipeline of the Stitch algorithm by Shelton *et al*. (2015) (https://github.com/i5K-KINBRE-script-share/Irys-scaffolding/blob/master/KSU_bioinfo_lab/assemble_XeonPhi/assemble_XeonPhi_LAB.md) was run on the online cluster zythos provided by the Pawsey Supercomputing Center, Western Australia. The first draft genome assembly of *T. subterraneum* (TSUd_r1.1) (Hirakawa *et al*., 2016) was used as the reference genome. Super‐scaffolds were generated, and BioNano IrysView was used to examine the new assemblies and the alignments between BioNano consensus maps and the *in* silico scaffolds. Based on these alignments, misassemblies were identified in TSUd_r1.1 (Figure 1b).

### Reference genome‐guided transcriptome sequence assembly

All RNASeq libraries were Illumina, San‐Diego, USA TruSeq adapter trimmed and quality trimmed (sliding window, minimum quality score: 20) using Trimmomatic v 0.36 (Bolger *et al*., 2014). Trimmed libraries were aligned to the advanced reference sequence using HISAT2 v 2.0.1 (insert size 0 to 1000) (Kim *et al*., 2015). The resulting SAM files were converted to BAM format using samtools v1.3 (Li *et al*., 2009).

BRAKER1 v 1.9 (Hoff *et al*., 2016) was used to predict genes using GeneMark‐ET v 4.32 (Lomsadze *et al*., 2014) and AUGUSTUS v 3.2.1 (Stanke *et al*., 2008) based on the RNASeq alignments.

### Functional annotation and classification of the transcriptome

The resulting predicted proteins were aligned to several databases using blastp v 2.2.31+ (Altschul *et al*., 1990) (minimum e‐value 1e^−10^). The database used were Swiss‐Prot and TrEMBL downloaded on March 13 2016 (Boeckmann *et al*., 2003) and all 2 542 385 predicted proteins downloaded from Phytozome v11 on 14 March 2016 (Goodstein *et al*., 2012). For each predicted protein, the hit with the highest score and lowest e‐value was chosen as annotation.

GO terms were assigned to proteins by manually transferring the GO terms for Swiss‐Prot IDs using the UniProt‐GOA database downloaded on 13 March 2016 (Huntley *et al*., 2015). KEGG K numbers were assigned to all predicted proteins using BLASTKOALA (taxonomy group: Plants, KEGG GENES database: family_eukaroytes) (Kanehisa *et al*., 2016).

### Tissue‐specific expression analysis

Tissue expression was estimated using kallisto v 0.42.4 (Bray *et al*., 2015). All genes with total transcripts per million (TPM) count below 1 or where more than two tissue types had a TPM of 0 were removed. To make log‐normalization possible, 0.25 was added to the remaining TPM values. Expression was log‐normalized and clustered into 12 clusters using Mfuzz v. 2.30.0 (Futschik and Carlisle, 2005) (m = 1.25).

### Hierarchical clustering of tissues based on global gene expression

Clustering was based on 100 genes with highest sum of all TPM (transcripts per million) of the five tissue types. The dendrogram of the selected genes [vertical axis] is visualized along with the expression patterns used to cluster the five tissue samples, i.e. root (RT), developing seed (DSD), leaf and petioles (LF_PT), stem and peduncles (ST_PD) and flower (FL) [horizontal axis]. There are five GO‐based clusters named G1, G2, G3, G4 and G5 that contain more than one gene. The GO clusters are separated by a horizontal bar in the heat map. Genes without annotations are omitted from the heat map. Over‐expressed genes are shown with red colour and under expressed genes with white or yellow colour, respectively (Figure 3).

### Genome structure and synteny analysis

Syntenic relationships between *M*. *truncatula* and *T. subterraneum* were calculated using SyMAP v4.2 (Soderlund *et al*., 2006, 2011). The *T. subterraneum* genome was mined for repeats using RepeatModeler and RepeatMasker (http://www.repeatmasker.org).

### Linkage map construction and quantitative trait locus (QTL) analyses

The linkage map was constructed using MultiPoint 3.3 (http://www.multiqtl.com/) as described in (Hirakawa *et al*., 2016). The complete set of 188 F2 lines of a biparental population 92S80 (cv. Woogenellup x cv. Daliak) phenotypic data as reported by Ghamkhar *et al*., 2011 was used for QTL screens for the following morphological and agronomic traits: levels of the oestrogenic isoflavones, formononetin (FO), genistein (GT) and biochanin A (BCA); days to first flowering (FT); leaf marks (LM); pigmentation of calyces (CP); hairiness of stems (SH); and hardseededness (HS). Levels of the isoflavones FO, GT and BCA were measured using the technique of Francis and Millington (1965). FT was measured as the number of days from sowing to appearance of the first flower. The morphological traits, LM, CP and SH, were scored 120 days after sowing, using the rating systems given in Nichols *et al*., (1996). Hardseededness was measured in the laboratory using the method of Quinlivan (1961).

QTL screens for the traits reported (Table 3) were conducted using an inclusive composite interval mapping (ICIM) approach implemented in QTL IciMapping v4.0 (Wang *et al*., 2014). Missing phenotypes were deleted using the ‘Deletion’ command in the software. The walking speed was set at 1 cm. A suitable probability for entering marker variables in stepwise regression was chosen so that the variation explained by the model approximated the trait heritability. The regression model was then used for background genetic variation control in the ICIM QTL mapping. The LOD was calculated using 1000 permutations, with a Type 1 error being 0.05, and significant QTLs were defined accordingly.

### Papilionoideae whole genome phylogenetic analyses

A phylogenetic tree was constructed, based on published predicted protein sequences for 13 legume genomes, including those of the newly annotated *Trifolium subterraneum* L. and *A. thaliana*. Mash v1.1 (Ondov *et al*., 2016) was used to calculate a distance matrix using k‐mer counting and pairwise mutation distance estimation, using the predicted amino acids from the published whole genome sequences for all the legumes, except *Cajanus cajan*, for which only the transcriptome assembly was used (as the whole genome assembly is not available). The tree was then constructed using UPGMA as implemented in the R‐package ‘phangorn’ v2.0.3. Shared and unique gene families were called using BLAST and OrthoMCL (Li *et al*., 2003). OrthomclToVenn (https://github.com/philippbayer/orthomcltovenn) was used to calculate the number of unique and shared genes between: (i) the Papilionoideae species and *A. thaliana*; (ii) the Millettioid, Galegoid and Dalbergioid clades; (iii) *A. thaliana* and the *Lotus*,* Trifolium*,* Medicago* and *Cicer* genera; and (iv) *T. subterranean* and *T. pratense*.

## Accession code

Advanced genome sequence assembly and annotation data have been made available (under embargoed) at https://zenodo.org/record/161479, DOI: http://doi.org/10.5281/zenodo.161479

## Author contributions

P.K. conceived and performed the research and wrote the manuscript with contributions from P.E.B, Z.M, J.V, Y.Y, R.A, D.E, J.B, P.N, J.D and W.E. The BioNano Irys^®^;System genome mapping experiments were led and designed by J.D and performed and analysed by Z.M, J.V and Y.Y. P.E.B and Y.Y did the bioinformatics analysis and helped with the figure preparations. All authors read the manuscript and approved the content.

## Supporting information


**Figure S1** The strategy and status of sequencing and assembly.
**Figure S2** Functional GO characterization of *Trifolium subterraneum L*. genes.
**Figure S3** Distribution of Kyoto Encyclopedia of Genes and Genomes (KEGG) pathways in the *Trifolium subterranean* annotation.
**Figure S4** Five tissue types of *Trifolium subterraneum L*. used for the transcriptome ATLAS.

**Table S1** Functional annotation of the predicted protein‐coding genes and transcripts.
**Table S2** Functional annotation of the predicted protein‐coding genes.
**Table S3** Functional composition of the assembled reference sequence *Trifolium subterraneum* L. genome assembly TSub_v2.0.
**Table S4** Non‐coding RNA genes in the advanced draft genome assembly.
**Table S5** Statistics of the completeness of the advanced genome Tsub_Refv2.0 assembly based using CEGMA pipeline.
**Table S6** List of 562 enriched GO terms among the unique 5648 GO terms, in the biological processes category using REVIGO web server.
**Table S7** List of 134 enriched GO terms among the unique 5648 GO terms, in the cellular processes category using REVIGO web server.
**Table S8** List of 316 enriched GO terms among the unique 5648 GO terms, in the molecular function category using REVIGO web server.
**Table S9** Hierarchical clustering of tissues based on global gene expression and GO terms.
**Table S10** Clustering of co‐expressed genes based on spatially enriched expression patterns.
**Table S11** List of GO terms for the leaf‐enriched Cluster 2, using REVIGO web server.
**Table S12** List of GO terms for the flower‐enriched Cluster 1, using REVIGO web server.
**Table S13** Number of putative genes‐encoding enzymes those mapped on KEGG pathways.
**Table S14** Distance matrix and pairwise mutation distance estimation calculated by Mash v1.1 (preprint: http://biorxiv.org/content/early/2016/04/19/029827) using the published whole genome sequences for all the legumes except the transcriptome assembly for *Cajanus cajan* as a whole genome assembly not available.
**Table S15** List of Trifolium‐specific genes among the galegoids identified using BLAST searches.
**Table S16** List of subterranean‐clover‐specific genes among the Trifoliums identified using BLAST searches.
**Table S17** List of red‐clover‐specific genes among the Trifoliums identified using BLAST searches.
**Table S18** Statistics of a SNP linkage map and numbers of anchored scaffolds.
**Table S19** High‐density SNP linkage map and anchoring information for *Trifolium subterraneum* L. genome assembly TSub_v2.0.
