# Supplementary material for: An advanced reference genome of Trifolium subterraneum L. reveals genes related to agronomic performance
Source: Plant Biotechnol J. 2017 Mar 23;15(8):1034–46. doi: 10.1111/pbi.12697 (PMC5506647; doi:10.1111/pbi.12697)
Supplement: Supplementary file 1 — Figure S1 The strategy and status of sequencing and assembly. Figure S2 Functional GO characterization of Trifolium subterraneum L. genes. Figure S3 Distribution of Kyoto Encyclopedia of Genes and Genomes (KEGG) pathways in the Trifolium subterranean annotation. Figure S4 Five tissue types of Trifolium subterraneum L. used for the transcriptome ATLAS. [file PBI-15-1034-s001.pdf]

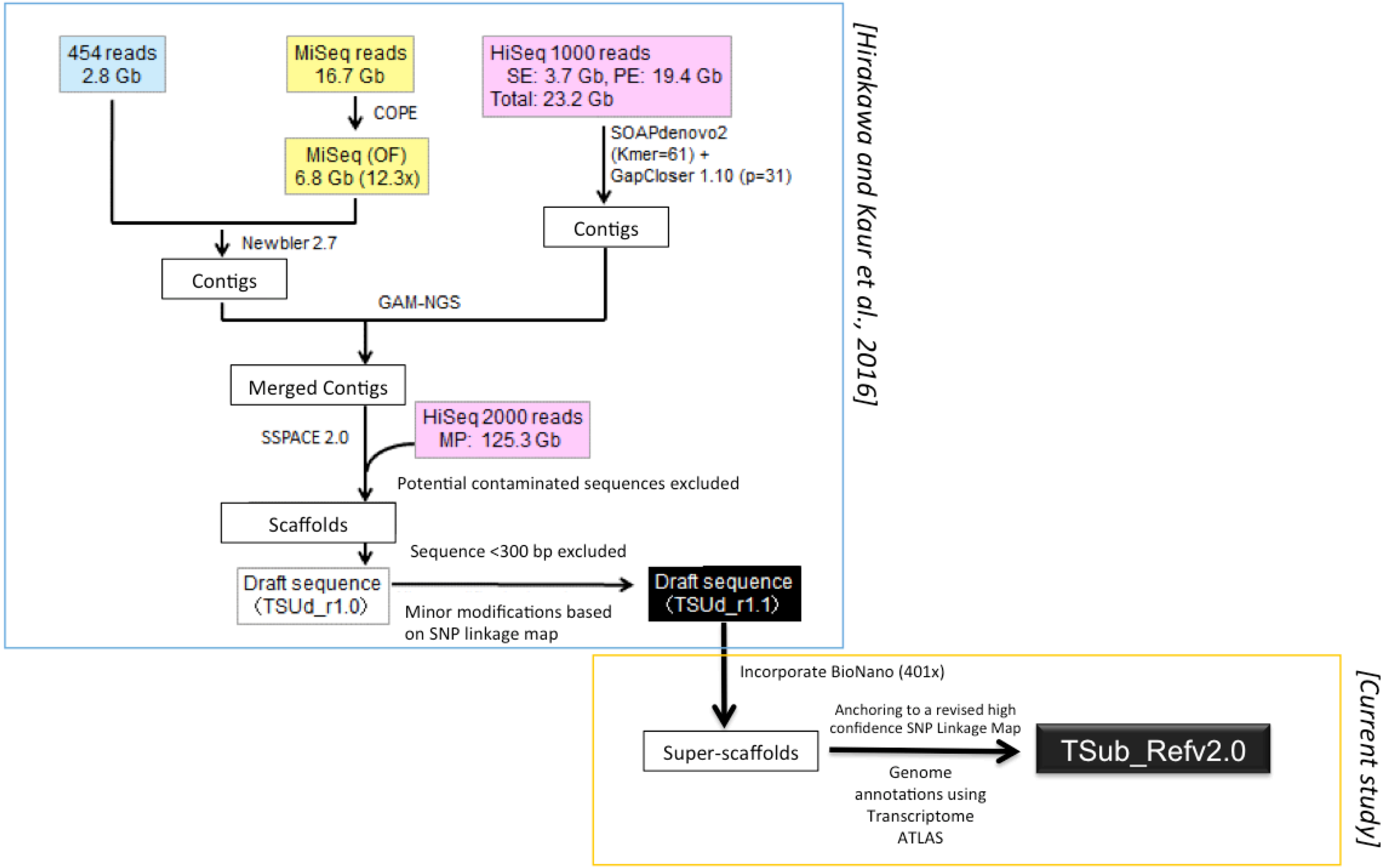

Fig S1. The strategy and status of sequencing and assembly

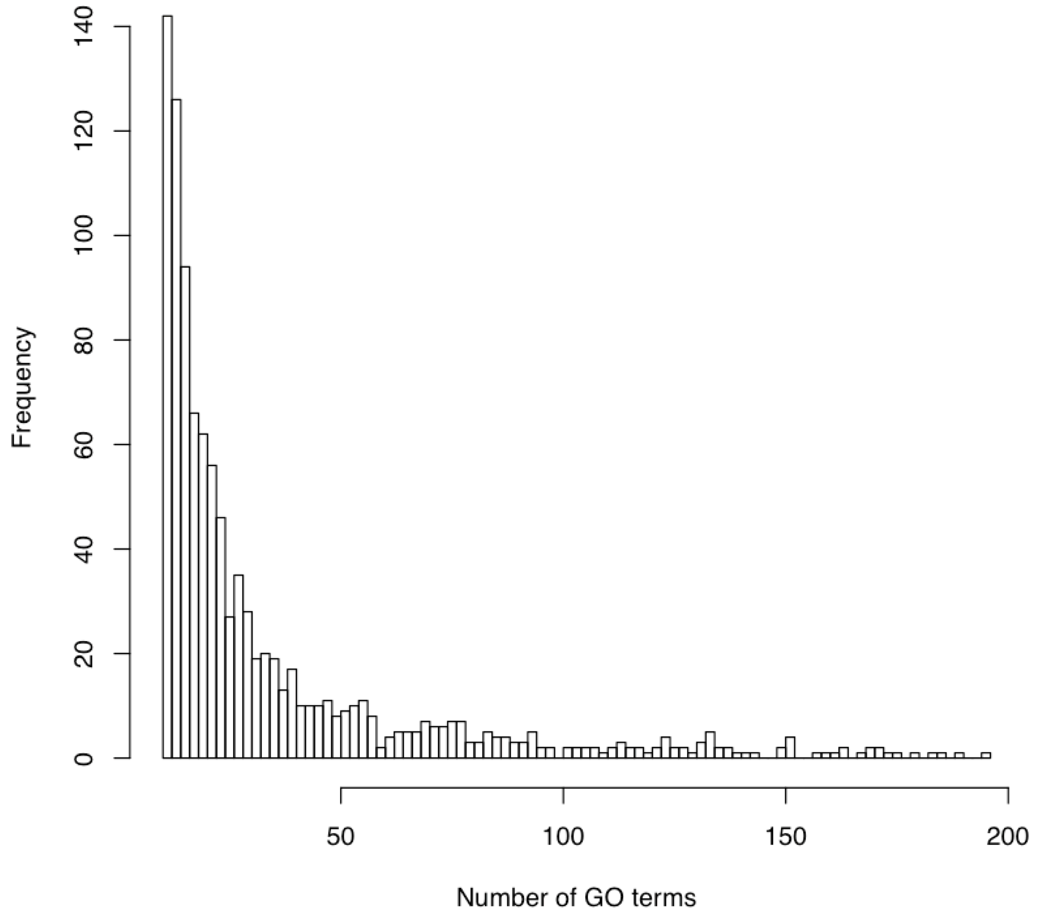

Fig S2. Functional GO characterisation of *Trifolium subterraneum L.* genes. Of all present GO terms including cellular components, biological processes, and molecular functions, 5648 are unique with a total sum of 131,324. There are 1,013 GO terms appearing between 50 and 200 times with a total sum of 35,383

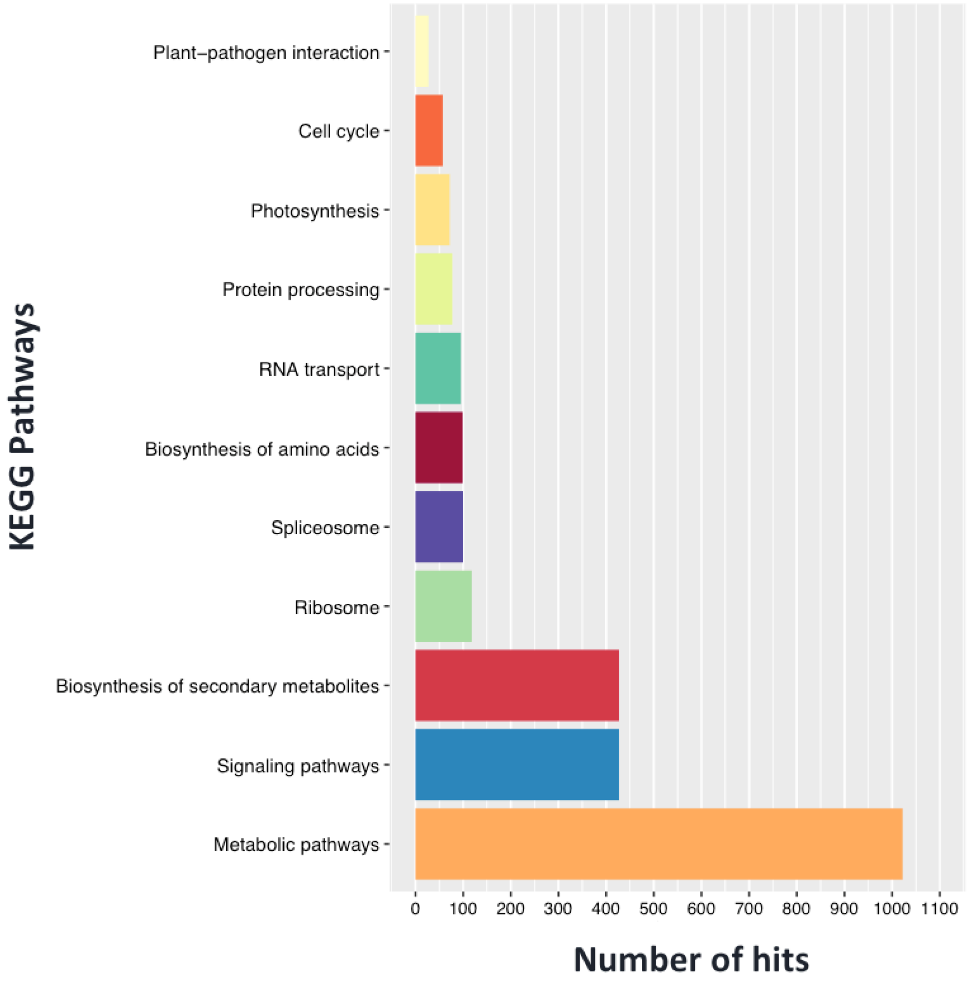

Fig S3. Distribution of Kyoto Encyclopedia of Genes and Genomes (KEGG) pathways in the *Trifolium subterranean* annotation. The KEGG Automatic Annotation Server was used to assign KEGG Orthology terms to the *T. subterranean* genes. The X- and Y-axes represent number of contigs and KEGG pathways, respectively. KEGG pathways containing ‘amino acids’, ‘biosynthesis’, and ‘signaling pathway’ were merged into one category ‘Biosynthesis of secondary pathways’, pathways containing ‘metabolism’ were merged into one category ‘Metabolic pathways’, pathways containing ‘signaling pathway’ were merged into the category ‘Signaling pathways’, pathways containing ‘photo’ were merged into one category ‘Photo synthesis’, and pathways containing ‘protein processing’ were merged into the category ‘Protein processing’

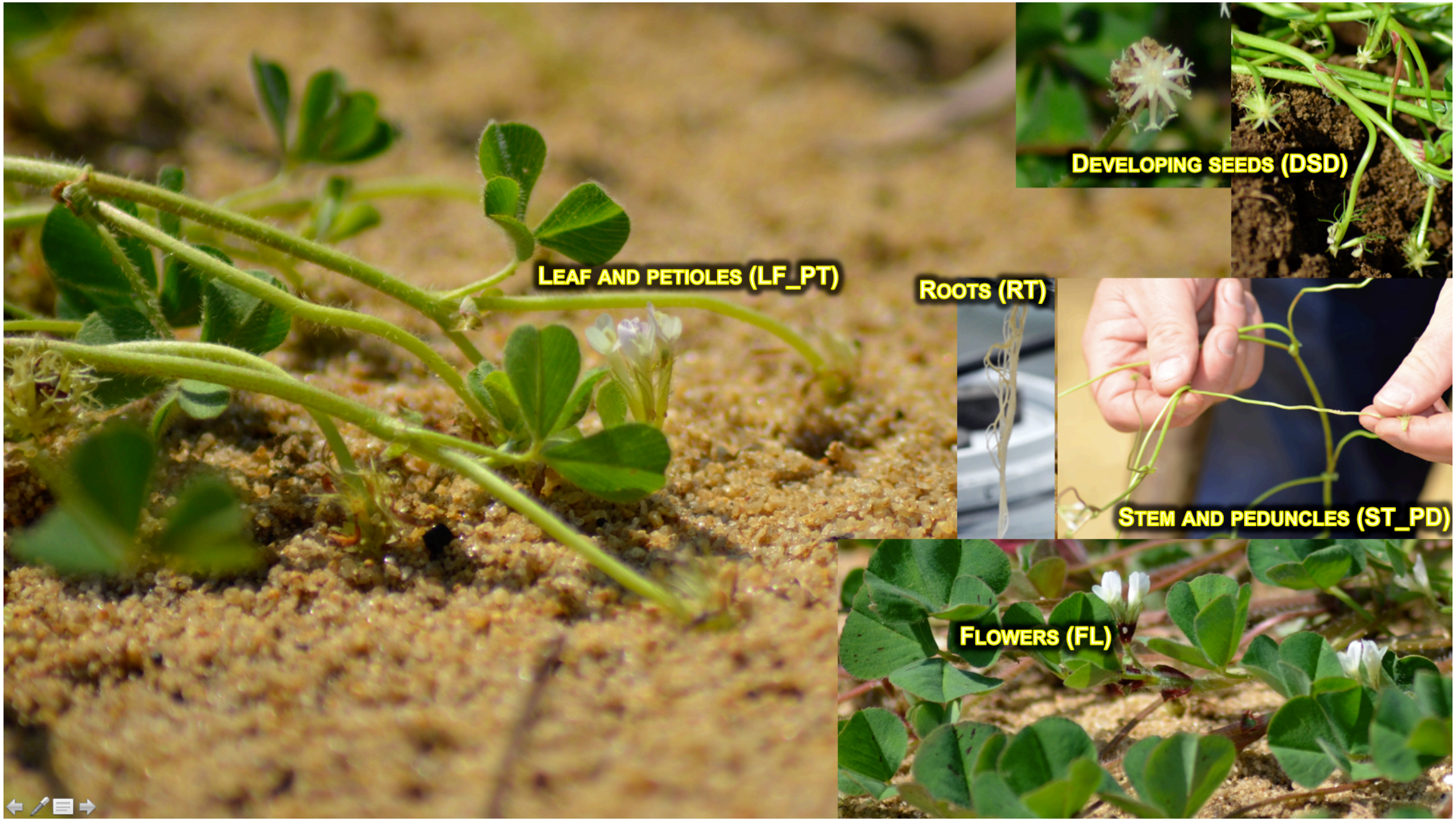

Fig S4. Five tissue types of *Trifolium subterraneum L.* used for the trancriptome ATLAS
